# Pan-Cancer Analysis of the Oncogenic and Immunological Role of Solute Carrier Family 6 Member 8 (SLC6A8)

**DOI:** 10.3389/fgene.2022.916439

**Published:** 2022-08-17

**Authors:** Xin Yang, Qiao Li

**Affiliations:** ^1^ Department of Surgery, People’s Hospital of Deyang City, Deyang, China; ^2^ Department of Pathology, People’s Hospital of Deyang City, Deyang, China

**Keywords:** *SLC6A8*, pan-cancer, prognosis, immunology, bioinformatics analysis

## Abstract

There is mounting evidence on the implication of *SLC6A8* in the initiation and progression of human cancers. However, a comprehensive understanding of the role of *SLC6A8* in pan-cancer remains elusive yet. Bioinformatics analysis was performed to investigate the expression and mutation profiles of *SLC6A8* in cancers, and the association of *SLC6A8* expression with cancer patients’ survival and immune cell infiltration. In general, *SLC6A8* is significantly upregulated across multiple cancers. *SLC6A8* expression was inconsistently prognostic in different types of cancer, albeit associated with favorable survival in the vast majority of cancers. The receiver operating characteristic curves showed that *SLC6A8* was relatively accurate in identifying possible cancer patients. The genetic alteration of *SLC6A8*, including mutation, amplification and deletion, was frequently present across various types of cancer. Mechanistically, *SLC6A8* might be involved in tumorigenesis through “carbon metabolism” and “HIF-1 signaling pathway.” Besides, *SLC6A8* expression had significant correlation with immune checkpoints genes and tumor-infiltrating immune cell abundances. The present study offers a novel insight into the roles of *SLC6A8* in the oncogenesis and development of multiple common cancers.

## Introduction

Despite the huge advances in medical resources and technology, the incidence and mortality of cancer are rapidly growing worldwide (Sung et al., 2021). Owing to the discovery and identification of star cancer-associated genes, targeted therapy and immunotherapy have transformed the treatment of cancer patients, leading to significantly prolonged survival and improving tolerance of cancer patients (Gotwals et al., 2017; Kennedy and Salama, 2020). For example, the implementation of first-generation tyrosine kinase inhibitors (TKIs) has significantly improved progression survival and overall survival as compared to traditional platinum-doublet chemotherapy in cancer patients harboring detectable sensitizing-EGFR mutations (Yoshioka et al., 2019). Nevertheless, the prognosis of most patients with advanced cancers remains poor due to the lack of available targetable driver mutations or emergence of therapy resistance (Gotwals et al., 2017; Camidge et al., 2019; Chai et al., 2022). Therefore, it is of urgent need to identify novel molecular targets that might play essential roles in the oncogenesis and malignant progression of cancers.

The solute carrier family 6 member 8 (*SLC6A8*) gene is located on chromosome X at the 28q locus and encodes creatine transporter protein which serves to transport creatine, a ubiquitous non-protein amino acid with pleiotropic effects (Bonilla et al., 2021), across cellular membranes (Joncquel-Chevalier Curt et al., 2015; Yıldız et al., 2020; Li et al., 2021). Initially, deletion or mutation of *SLC6A8* was found to be associated with creatine transport deficiency (CTD) such as intellectual disability and verbal dyspraxia (Battini et al., 2007). Lately, it has been reported that *SLC6A8* is implicated in the malignant progression of various tumor. For example, therapeutic targeting of *SLC6A8* suppresses colon cancer progression by modulating creatine abundances (Kurth et al., 2021). SLC6A8 is required for the malignant progression of NSCLC via activating Notch signaling pathway (Feng et al., 2021). Moreover, deficiency in SLC6A8-mediated creatine transport is reported to impair immunogenic function (Di Biase et al., 2019). For instance, loss of *SLC6A8* weakens TCR-mediated activation of mechanistic target of rapamycin complex 1 (mTORC1) signaling, resulting in hampered CD8^+^ T cell immunity (Samborska et al., 2022). Ablation of SLC6A8 reprogrammed macrophage polarization via regulating cytokine responses, contributing to altered immune responses *in vivo* (Ji et al., 2019). However, the oncogenic roles of *SLC6A8* across different cancers remain unknown. Considering the involvement of *SLC6A8* in multiple physical and pathological processes (Salomons et al., 2003), we sought to comprehensively explore the roles this gene plays in cancers through bioinformatics analysis.

Herein, we found that *SLC6A8* expression was differentially expressed in 22 types of cancer while no significant difference was observed for *SLC6A8* expression in the remaining 10 types of cancer. In addition, *SLC6A8* was found to be a significant prognostic and diagnostic factor in various cancers. Moreover, *SLC6A8* gene was often altered, with amplification, mutation and deletion being the dominant alteration types. Analysis of possible pathways involving *SLC6A8* showed that *SLC6A8* was mainly enriched in “Carbon metabolism,” “HIF-1 signaling pathway,” and “Glycolysis/Gluconeogenesis” pathways. Finally, the immunological roles of *SLC6A8* in various cancers were evaluated.

## Materials and Methods

### Expression Analysis

The Cancer Genome Atlas (TCGA, http://cancergenome.nih.gov) is a landmark cancer genomics program that sequenced and characterized the molecular landscape of tumors from 11,160 patients across 33 cancer types (Liu et al., 2018). Gene Expression Profiling Interactive Analysis 2 (GEPIA2, http://gepia2.cancer-pku.cn) is a valuable and highly cited resource for gene expression analysis based on tumor and normal samples from the TCGA and the GTEx databases (Tang et al., 2019). The differential expression of *SLC6A8* between tumor tissues and their normal counterparts were analyzed based on data from TCGA and GEPIA2 databases. *p* value <0.05 and |Log_2_FC| > 1 were set as the cutoffs of significantly differential expression of *SLC6A8*.

### Survival Analysis and Receiver Operating Characteristic Analysis

PrognoScan (http://www.prognoscan.org/) is an online tool for evaluating the biological relationship between gene expression and patient prognosis based on publicly available cancer microarray datasets with clinical annotation (Mongre et al., 2019). Using the database, we conducted univariate cox regression analysis to evaluate the prognostic value of *SLC6A8* in pan-cancer. *p* value <0.05 was considered to be statistically significant.

ROC curves were plotted to characterize the accuracy of *SLC6A8* expression to discriminate between patients with or without cancer based on data from TCGA database.

### Analysis of Mutation Landscape of *SLC6A8*

The cBio Cancer Genomics Portal (cbioportal, http://cbioportal.org) is an open-access resource for interactive exploration of multidimensional cancer genomics datasets including mutation data (Cerami et al., 2012). *Via* cbioportal database, the alteration types and sites of *SLC6A8* in pan-cancer were identified based on the data of 10,967 samples from TCGA database. The filtering criteria of associations were set as following: Correlation ≥0.4, −Log10(p) ≥2, Max results = 200.

### Protein-Protein Interaction Analysis and Pathway Enrichment Analysis

The Search Tool for the Retrieval of Interacting Genes/Proteins (STRING, https://string-db.org) aims to integrate all known and predicted associations between proteins (Szklarczyk et al., 2021). The interacting network of *SLC6A8* protein with other associated proteins were visualized through STRING database. The cutoff value of interaction score was set as 0.15. The venn diagram was utilized to display intersecting members of STRING and GEPIA2.

For pathway enrichment analysis, both Kyoto Encyclopedia of Genes and Genomes (KEGG) pathway analysis and Gene Ontology (GO) enrichment analysis were conducted to visualize the cellular pathways that *SLC6A8* was most significantly related to. The cutoff *Q* value and *p* value were set as 0.05 and 0.01, respectively.

### Immunity-Related Analysis

Tumor Immune Estimation Resource (TIMER2, http://timer.cistrome.org/) web server provides comprehensive analysis and visualization of the functions of tumor infiltrating immune cells by integrating multiple algorithms that have been designed to estimate immune infiltration (Li et al., 2017). We analyzed immune infiltration in different cancer types based on the TCGA datasets through TIMER2 database. Besides, the associations between *SLC6A8* expression and microsatellite instability (MSI), and tumor mutation burden (TMB) were assessed using SangerBox platform (http://sangerbox.com/Tool), an online platform for analysis of TCGA data. Pearson correlation coefficient was employed to analyze the correlation between *SLC6A8* expression and other genes.

## Results

### Pan-Cancer Expression Profile of *SLC6A8*

**FIGURE 1:**
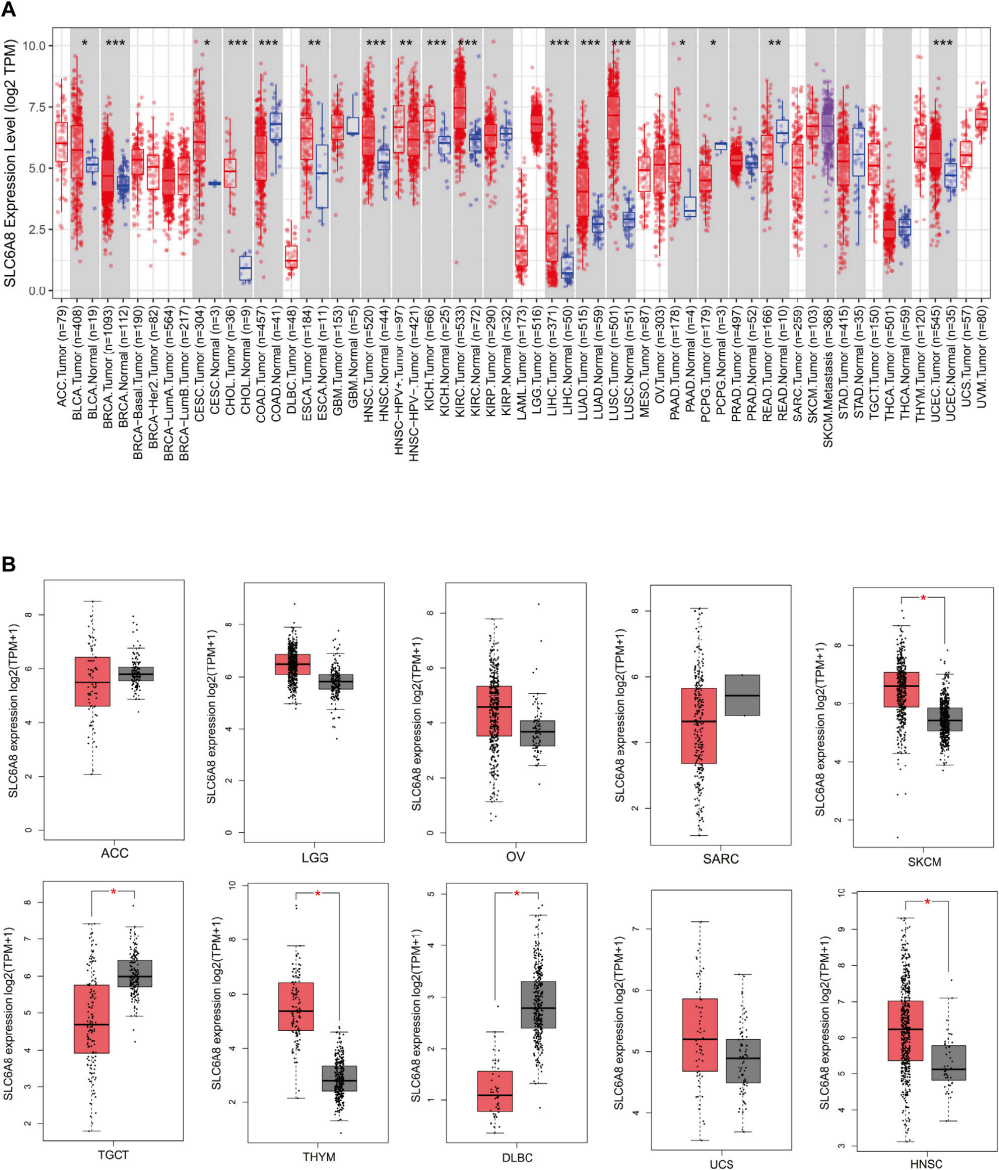
*SLC6A8* expression profiles in cancers. **(A)** Pan-cancer differential-expression data for *SLC6A8* between tumor tissues and paired normal tissues based on TCGA data. **(B)** Analysis of *SLC6A8* expression levels in tumor tissues versus corresponding normal tissues via GEPIA2 platform. *p < 0.05, **p < 0.01, ***p < 0.001.

To investigate *SLC6A8* expression pattern in various human cancers, we analyzed the transcriptome data acquired from TCGA database. The results showed that *SLC6A8* was significantly upregulated in 13 types of cancer including bladder urothelial carcinoma (BLCA), breast invasive carcinoma (BRCA), cervical squamous cell carcinoma and endocervical adenocarcinoma (CESC), cholangiocarcinoma (CHOL), esophageal carcinoma (ESCA), head and neck squamous cell carcinoma (HNSC), kidney chromophobe (KICH), kidney renal clear cell carcinoma (KIRC), liver hepatocellular carcinoma (LIHC), lung adenocarcinoma (LUAD), lung squamous cell carcinoma (LUSC), pancreatic adenocarcinoma (PAAD), and uterine corpus endometrial carcinoma (UCEC). Of note, *SLC6A8* expression in LUAD, LUSC, and CHOL was remarkably higher than in normal counterparts. Interestingly, 3 types of cancer, including colon adenocarcinoma (COAD), pheochromocytoma and paraganglioma (PCPG) and Rectum adenocarcinoma (READ), exhibited significantly lower *SLC6A8* expression as compared to their respective normal tissues (Figure 1A). For the 11 types of cancer in which *SLC6A8* expression in their corresponding normal tissues was lacking, the GTEx dataset in GEPIA2 website was used for comparison of *SLC6A8* expression between tumor and normal tissue. According to the results, *SLC6A8* expression was found to be significantly upregulated in skin cutaneous melanoma (SKCM), thymoma (THYM), and head and neck squamous cell carcinoma (HNSC), whereas the downregulation of *SLC6A8* was found in testicular germ cell tumors (TGCT) and lymphoid neoplasm diffuse large B-cell lymphoma (DLBC) (Figure 1B). Taken together, *SLC6A8* was aberrantly expressed in diverse cancers.

### Pan-Cancer Analysis of the Prognostic and Predictive Value of *SLC6A8*

**FIGURE 2:**
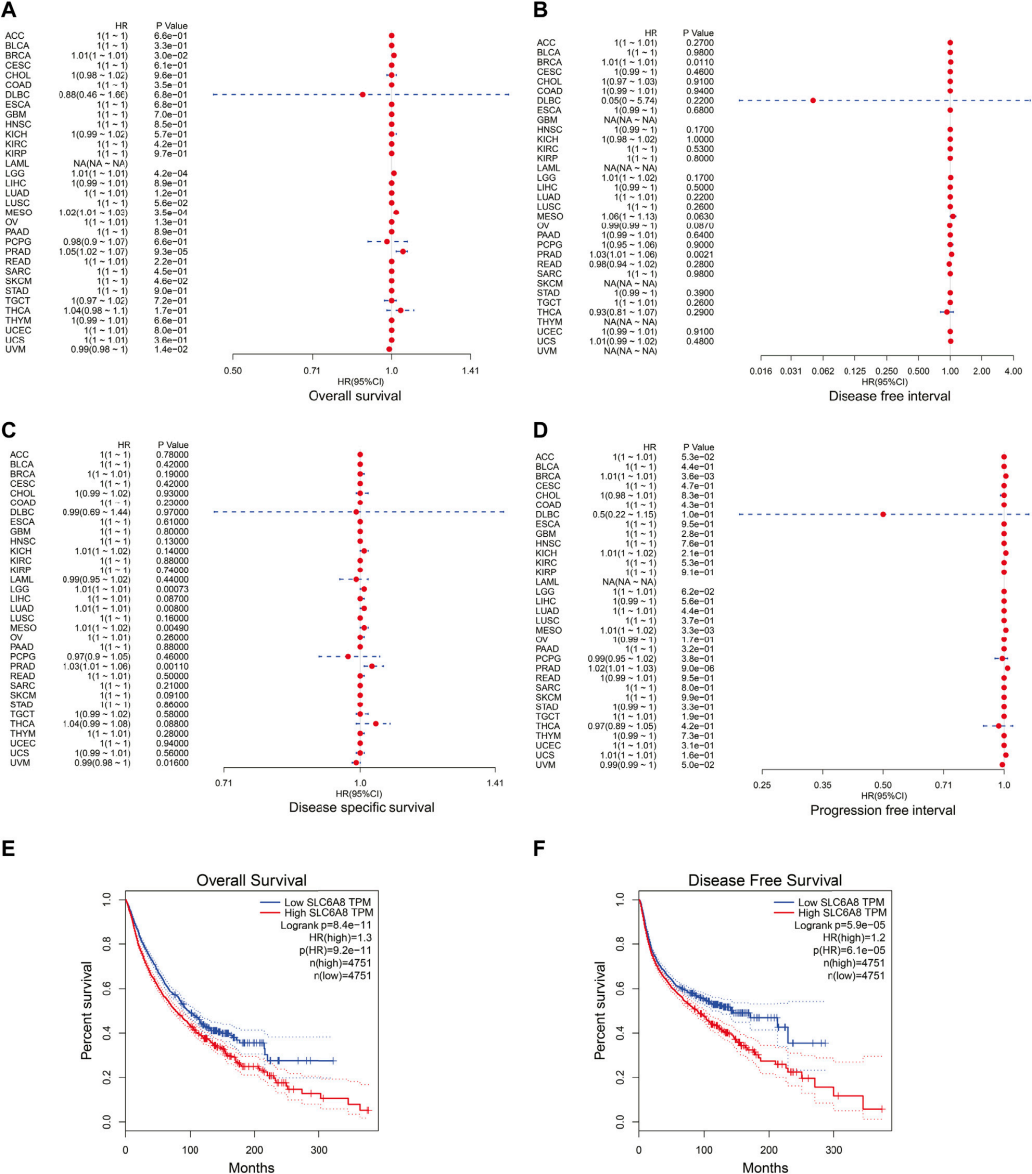
Correlation between *SLC6A8* expression and cancer patients’ survival. **(A**–**D)** Forest plots of hazard ratios of *SLC6A8* in different cancers regarding overall survival **(A)**, disease-free interval **(B)**, disease specific survival **(C)**, and progression free interval **(D)**. **(E)** and **(F)** Kaplan-Meier survival curves of overall survival **(E)** and disease-free survival **(F)** according to *SLC6A8* expression in pan-cancer according in GEPIA2. *p < 0.05, **p < 0.01, ***p < 0.001.

**FIGURE 3:**
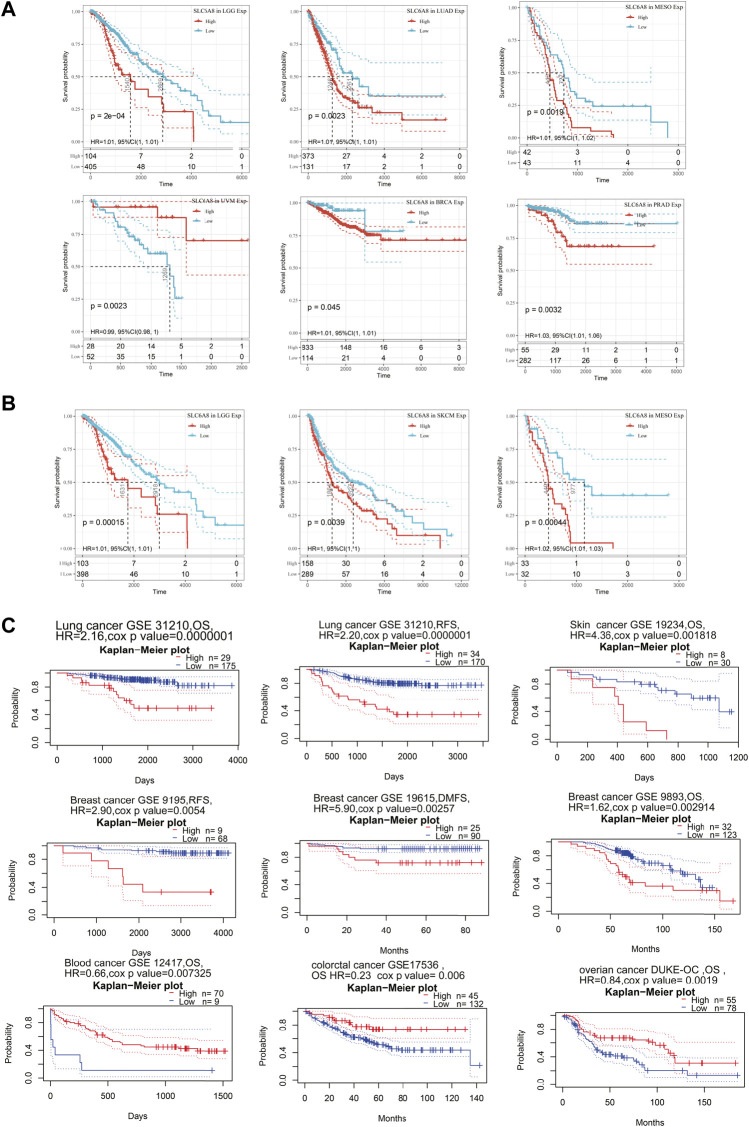
Survival analysis based on *SLC6A8* expression in cancers. **(A)** and **(B)** Survival curves with significance of overall survival **(A)** and disease-free survival **(B)** based on *SLC6A8* expression downloaded from TCGA. **(C)** Kaplan–Meier plots according to prognoscan database. *p < 0.05, **p < 0.01, ***p < 0.001.

In order to assess the relationship between *SLC6A8* expression and patients’ prognoses in 33 types of cancer, univariate cox regression analysis was performed based on data from TCGA. *SLC6A8* expression was significantly associated with the overall survival (OS) of BRCA, LGG, mesothelioma (MESO), and prostate adenocarcinoma (PRAD), disease free interval (DFI) of BRCA and PRAD, disease specific survival (DSS) of LGG, LUAD, MESO, PRAD, and uveal Melanoma (UVM), progression free interval (PFI) in BRCA, MESO, and PRAD (Figures 2A–D). Kaplan–Meier survival curves of pan-cancer patients showed an adverse pan-cancer prognostic association of *SLC6A8* expression (Figures 2E,F). Specifically, *SLC6A8* expression was negatively associated with overall survival in LGG, LUAD, MESO, BRCA, and PRAD while positively associated with OS in UVM (Figure 3A). Consistently, *SLC6A8* expression was negatively associated with the disease-free survival in LGG, MESO and, unexpectedly, SKCM (Figure 3B). Furthermore, we investigated the association between *SLC6A8* expression and the prognosis of cancer patients through bioinformatics analysis of the GEO dataset via the Prognoscan database. Notably, upregulation of *SLC6A8* was found to be inversely related to the overall survival in cancer of lung, breast and skin, relapse free survival (RFS) in cancer of the lung and breast, and distant metastasis-free survival (DMFS) in breast cancer. However, *SLC6A8* expression was favorably related to the OS in blood, colorectal and ovarian cancer (Figure 3C).

**FIGURE 4:**
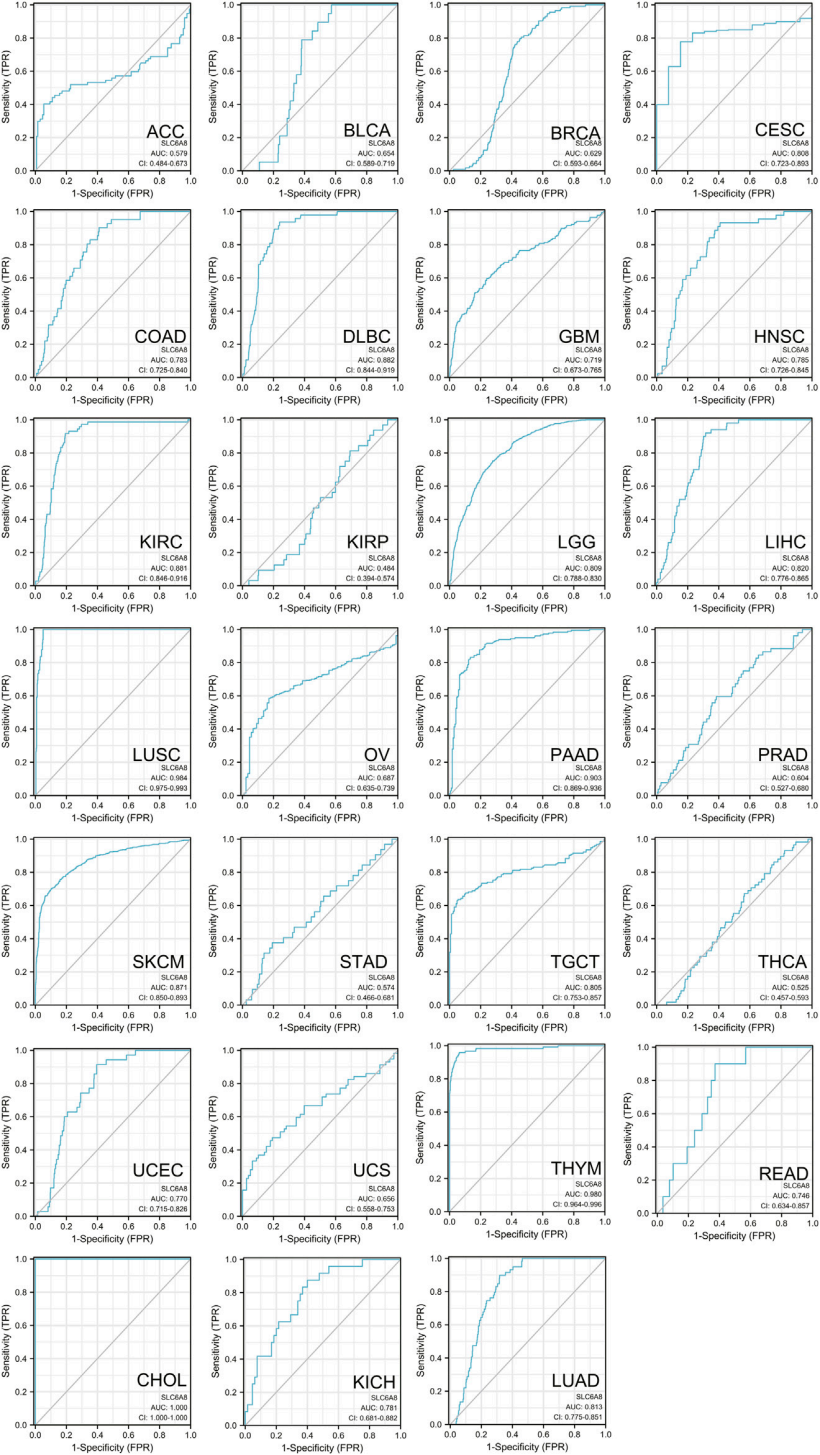
ROC analysis showing predictive potential of *SLC6A8* in pan-cancer.

Besides, ROC curve was used to evaluate the clinical prediction accuracy of *SLC6A8* in 27 cancer types. Remarkably, *SLC6A8* was showed to be able to discriminate cancer patients from those without cancer in 26 of 27 types of cancer (AUC >0.5) (Figure 4). Specifically, the AUC value of three fourths of cancer exceeded 0.7, meaning that *SLC6A8* was relatively accurate in identifying possible cancer patients. Taken together, *SLC6A8* might be a potential prognostic and predictive factor in various cancers.

### Pan-Cancer Analysis of the Mutation Landscape of *SLC6A8*

**FIGURE 5:**
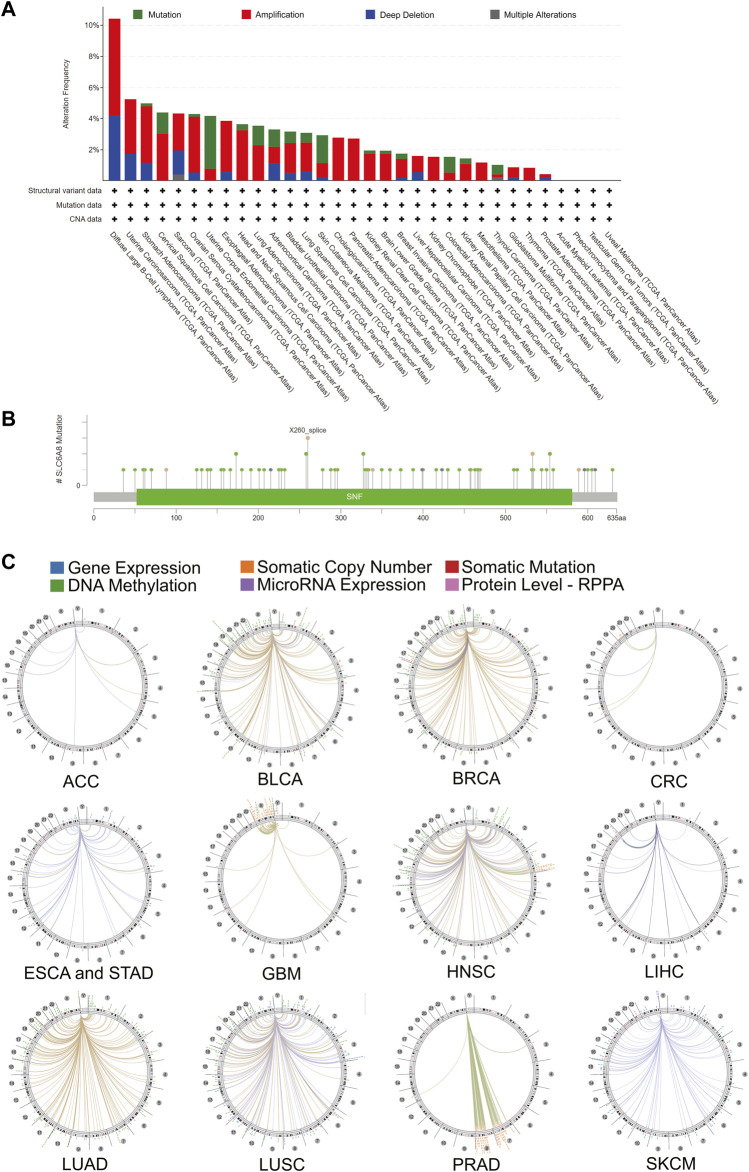
Mutational landscape of SLC6A8 and its association with genome in cancers. **(A)** *SLC6A8* gene alteration frequency across cancers. **(B)** Mutation sites of *SLC6A8* in pan-cancer. **(C)** The correlation between *SLC6A8* and other signatures of genome based on TCGA data.

To investigate the genetic alteration of *SLC6A8* across different types of cancer, bioinformatics analysis was performed based on the data of 10,967 samples from TCGA database via cBioPortal database. As shown in Figure 5A, “amplification” represented the primary mutation type for the majority of cancer types. And the highest alteration frequency (>10%) occurred in DLBC, also with “amplification” as the dominant type. PRAD showed the lowest alteration frequency of <1%. Notably, Mutation was the dominant type of genetic alteration in UCEC. Moreover, among the 72 mutations that are distributed between amino acids 52 and 581, X260 splice (from SKCM, STAD, and BRCA samples) was found to be the site with the highest mutation frequency (Figure 5B). The details of all *SLC6A8* mutation profiles were summarized in Supplementary Table S1. Besides mutation, deletion and amplification, we further investigated the association between *SLC6A8* and other genomic signatures, including gene expression, somatic copy number, somatic mutation, DNA methylation microRNA expression and protein level-RPPA. As illustrated by the circos plots, *SLC6A8* was associated with other genomic signatures in ACC, BLCA, BRCA, CRC, ESCA&STAD, GBM, HNSC, LIHC, LUAD, LUSC, PRAD, and SKCM (Figure 5C). Thus, *SLC6A8* is a potential factor contributing to modulation of the mutational landscape in various cancers.

### Pan-Cancer Analysis of the Biological Functions of *SLC6A8*

**FIGURE 6:**
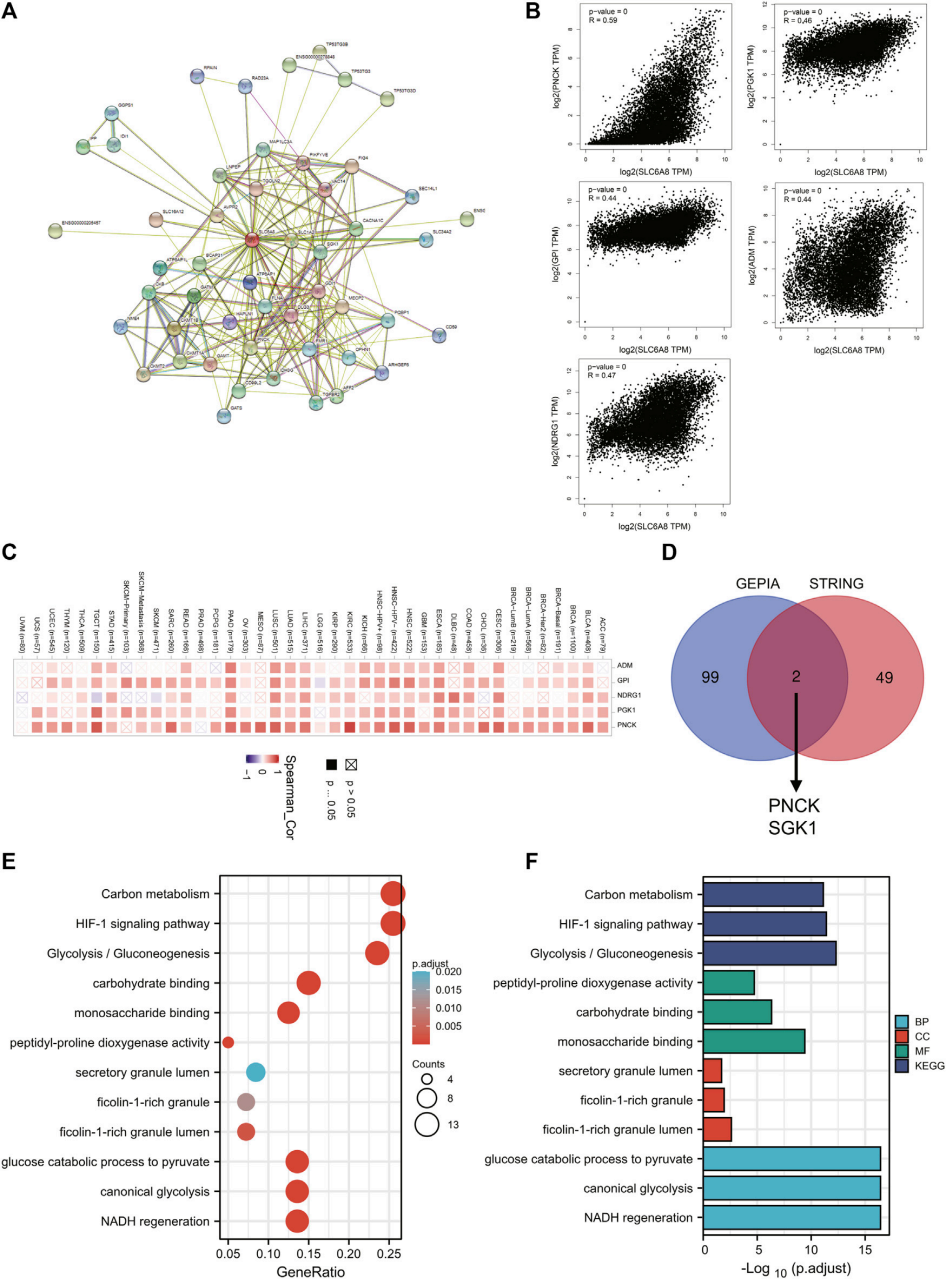
Analysis of pathways enriched for *SLC6A8* in cancers. **(A)** Protein-protein interaction network of SLC6A8 was determined via STRING database. **(B)** Pearson correlation analysis of SLC6A8 expression and its top 5 related genes according to GEPIA2. **(C)** The expressions of the 5 genes that are most related to *SLC6A8* expression in detailed cancer types were illustrated by the heatmap. **(D)** The venn diagram showing the common members of GEPIA2-based SLC6A8-associated genes and STRING-based SLC6A8-interacting proteins. **(E)** and **(F)** KEGG pathway **(E)** and GO **(F)** enrichment analysis based on *SLC6A8*-related genes (proteins).

To explore the mechanisms underlying the oncogenic role of *SLC6A8*, pathway enrichment analyses were conducted following identification of genes and proteins that were associated with *SLC6A8*. As shown in Figure 6A, a total of 49 *SLC6A8*-interacting proteins were identified. Moreover, we analyzed the top 100 genes that were associated with *SLC6A8* expression based on tumor data from GEPIA2. The expression of *SLC6A8* exhibited the strongest positive correlation with that of ADM, GPI, NDRG1, PGK1, and PNCK (Figure 6B), and their correlation with *SLC6A8* expression in distinct cancer types were illustrated by the heatmap, respectively (Figure 6C). Of note, interaction analysis of STRING-based interacting proteins and GEPIA-based correlated genes of *SLC6A8* identified 2 common members (e.g., PNK1 and SGK1) (Figure 6D).

To investigate the effects of *SLC6A8* on cellular pathways and functions, we conducted KEGG and GO enrichment analysis based on *SLC6A8*-associated genes in tumor. KEGG analysis indicated that “Carbon metabolism,” “HIF-1 signaling pathway” and “Glycolysis/Gluconeogenesis” were three most enriched pathways (Figure 6E). Additionally, GO molecular function analysis revealed that *SLC6A8* was associated to “peptidyl-proline dioxygenase activity,” “carbohydrate binding” and “monosaccharide binding” (Figure 6F). Thereof, these results suggest that *SLC6A8* might influence tumor progression via reshaping cellular metabolism.

### Pan-Cancer Analysis of the Relationship Between Immune Landscape and *SLC6A8* Expression

**FIGURE 7:**
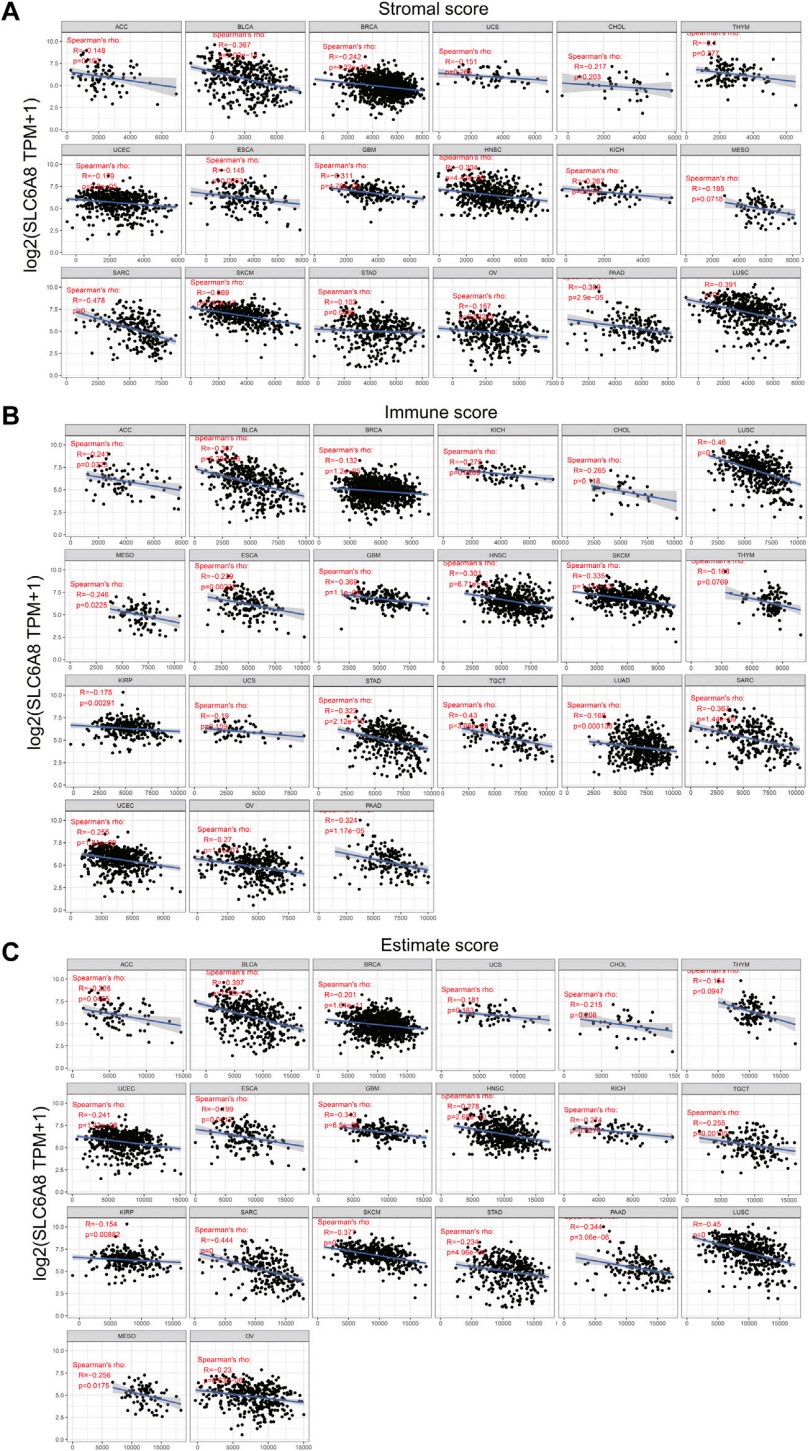
*SLC6A8* expression was associated with immune conditions. **(A)**–**(C)** *SLC6A8* expression was negatively related with immune score **(A)**, stromal score **(B)** and estimate score **(C)** in various cancers.

**FIGURE 8:**
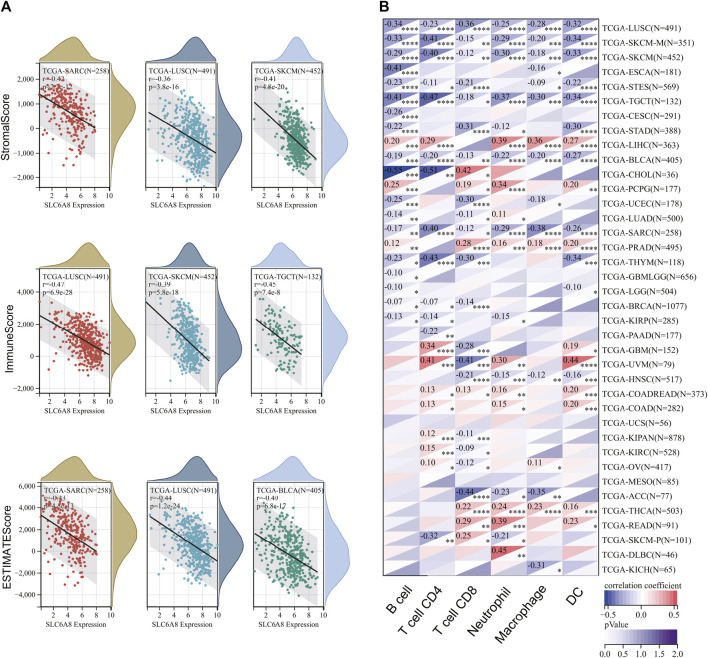
The correlation between *SLC6A8* and immune status in different cancers. **(A)** The negative correlation between *SLC6A8* and stromal, immune, and estimate scores is the strongest in LUSC. **(B)** Analysis of associations between *SLC6A8* expression and scores of tumor-infiltrating immune cells in TIMER database.

**FIGURE 9:**
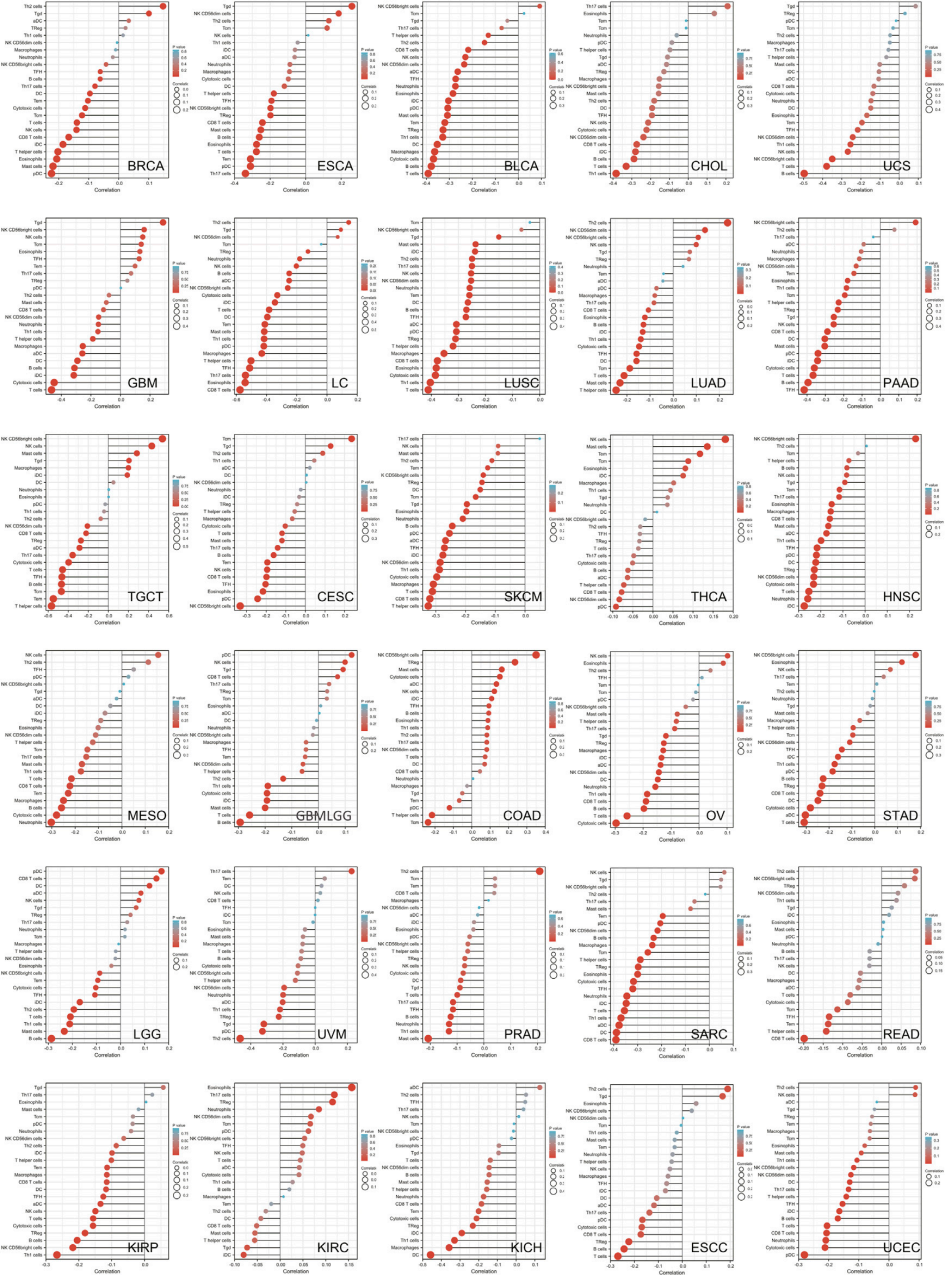
The stick charts showing the relationship between SLC6A8 expression and levels of diverse tumor-infiltrating immune cells in various cancers.

To explore the relationship between tumor immune microenvironment and *SLC6A8*, we applied R software package to evaluate the stromal, immune, and estimate scores (Petitprez et al., 2020). *SLC6A8* expression was negatively correlated with stromal, immune, and estimate scores in the majority of cancers analyzed (Figures 7A–C). Notably, the negative correlation between *SLC6A8* and all three scores was the strongest in LUSC (Figure 8A). Next, we analyzed the relationship between *SLC6A8* expression and scores of tumor-infiltrating immune cells based on data from TIMER database, and found that the most negatively related tumors were LUSC, SKCM and TGCT (Figure 8B). In addition, the correlation of *SLC6A8* expression with various tumor-infiltrating immune cells was further explored in 30 types of cancer (Figure 9). *SLC6A8* expression was found to be negatively associated with the majority of tumor-infiltrating immune cells in most of the cancers such as BRCA, UCS, LUSC, SKCM, KIRP, and KICH. Collectively, those results indicate that *SLC6A8* might be involved in the regulation of tumor immune microenvironment.

### Pan-Cancer Analysis of the Association Between *SLC6A8* Expression and Immunotherapy

**FIGURE 10:**
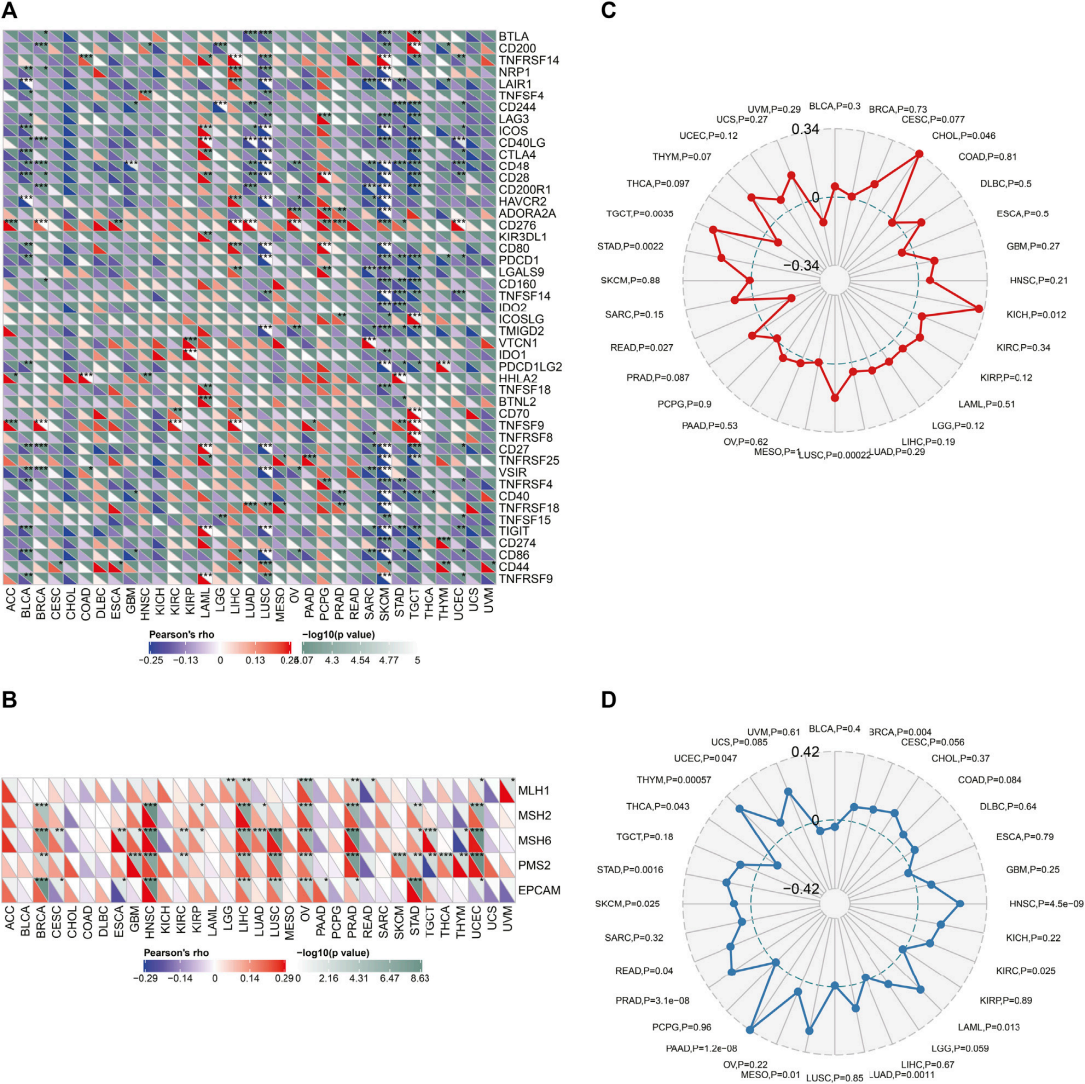
Relationship between *SLC6A8* and response to immunotherapy. **(A)** Correlations between expression of *SLC6A8* and representative immune checkpoint genes in different cancers. **(B)** Correlations of *SLC6A8* and 5 component genes of MMR system across cancers. **(C)** and **(D)** Correlations of *SLC6A8* with MSI **(C)** and TMB **(D)**. *p < 0.05, **p < 0.01, ***p < 0.001.

To evaluate the association between *SLC6A8* expression and immunotherapy, we analyzed the correlation between *SLC6A8* expression and some representative immune checkpoints genes (Dijkstra et al., 2016). As displayed in Figure 10A, the expression of *SLC6A8* was positively correlated with that of immune checkpoints genes in LAML, PCGC, and LIHC while negatively correlated with them in BLCA, SKCM and LUSC. Notably, CD276 expression was remarkably associated with *SLC6A8* in 11 of 32 types of cancer (Figure 10A). MutL homologue 1 (MLH1), mutS homologue 2 (MSH2), mutS 6 (MSH6), postmeiotic segregation increased 2 (PMS2), and epithelial cell adhesion molecule (EPCAM) are the key components of the DNA mismatch repair (MMR) system, deficiency in which accounts for a significant proportion of human cancers by inducing instability in genome (McGrail et al., 2020). As illustrated by the heatmap, *SLC6A8* expression had significantly positive correlation with these MMR genes in 8 types of cancer, including BRCA, HNSC, LIHC, LUSC, OV, PRAD, STAD, and UCEC (Figure 10B). MSI and TMB are both considered to be valuable predictive biomarkers of patients’ response to immune checkpoints inhibitors (ICIs) (Shum et al., 2022). *SLC6A8* expression was significantly higher in MSI-high tumor tissues than MSI-low ones in KICH, LUSC, STAD and TGCT (Figure 10C). Additionally, *SLC6A8* expression was also positively associated with TMB in BRCA, HNSC, KIRC, LAML, LUAD, MESO, PAAD, PRAD, SKCM, STAD, THCA, THYM, and UCEC (Figure 10D). Nevertheless, all statistically significant coefficients of *SLC6A8* with MSI/TMB were below 0.4, indicating that *SLC6A8* was a weak biomarker to predict response to immunotherapy.

## Disscussion

The *SLC6A8* gene is located on the long arm of chromosome X and contains 14 exons. By acting as a plasma membrane transporter, SLC6A8 protein enables cells to import extracellular creatine or its precursor, guanidinoacetate, into cells against steep concentrations, thus maintaining the intracellular creatine levels (Jomura et al., 2022). Recently, SLC6A8 was reported to be involved in the malignant progression of several cancers, including NSCLC, CRC, and HCC via regulation of key signaling pathways. Our previous works have demonstrated that SLC6A8 could promote the survival of triple-negative breast cancer cells by maintaining intracellular creatine levels (Li et al., 2021). Nevertheless, analysis of the role of *SLC6A8* in pan-cancer remains unexplored. Here, we conducted a pan-cancer bioinformatics analysis of the expression and mutation profile, and clinical significance of *SLC6A8* and its potential effect on malignancy.

In this work, we sought to thoroughly investigate the function of *SLC6A8* in various cancers by integrating complementary bioinformatics methods and public databases. The results of gene expression analysis showed that *SLC6A8* was upregulated in most types of the cancers while downregulated in a few types of cancers as compared to their corresponding normal counterparts (Figures 1A,B). According to the results of the survival analysis, *SLC6A8* expression predicted poor overall survival and disease-free survival of cancer patients (Figures 2E,F). Intriguingly, *SLC6A8* expression had positive correlation with the overall survival of patients diagnosed with blood, colorectal and ovarian cancer (Figure 3C). ROC curve analysis results indicated that *SLC6A8* expression was able to discriminate cancer patients with relatively good accuracy (AUC>0.5) (Figure 4). Our findings unprecedentedly revealed the potential prognostic and diagnostic value of *SLC6A8* in pan-cancer.

Cancer immunotherapies have been proved to induce durable responses in multiple malignancies, and thus have transformed the treatment landscapes for a subset of cancers: NSCLC, GC, and BLCA, to name a few (Borcoman et al., 2019). One of the vital strategies of immunotherapy is to overcome cancer immune evasion by modulating the functionality of immune cells (Marin-Acevedo et al., 2021). For instance, GBM stem cells (GSCs) escape immune clearance *via* increasing recruitment of tumor-associated macrophages (Gangoso et al., 2021). Accumulating studies have illustrated that *SLC6A8* was intricately implicated in cell immunity by regulating immune cells including T cells and macrophages (Ji et al., 2019; Peng et al., 2021). Herein, we found that *SLC6A8* expression was negatively correlated with the stromal, immune, and estimate score in the majority of cancers (Figures 7, 8). Besides, *SLC6A8* had negative correlation with the levels of various tumor-infiltrating immune cells in pan-cancer (Figure 9). These results collectively indicated that *SLC6A8* might be associated with enhanced immunosuppressive tumor microenvironment. Notably, ICIs have revolutionized the paradigm in cancer immunotherapy (Nakajima and Nakatsura, 2021). Mechanistically, ICIs could competitively interact with immune checkpoint proteins to relieve cancer-mediated inhibition of T-cell function (Liu et al., 2020). Our study firstly uncovered the association between *SLC6A8* and several common biomarkers of response to ICIs (e.g., levels of checkpoint genes, MSI, and TMB) in multiple cancers (Figure 10). These results suggest that *SLC6A8* expression is a potential biomarker of patient prognosis and response to immunotherapy.

In spite of the fact that numerous public databases have been integrated to clarify the role of *SLC6A8* in pan-cancer, several limitations to the present work should be pointed out. First, simple combination of sequencing and microarray data across different databases for analysis of tumor information might bias the data and reduce the reliability of the analysis due to a lack of analysis of data covering cellular-level information. Second, all the conclusions in this study were solely based on the results of bioinformatics analysis. Further *in vitro*/vivo experiments are necessarily needed to validate the results. Our next step is to confirm and elucidate the role of *SLC6A8* across different cancers experimentally. Finally, according to the results of the study, *SLC6A8* exhibited conflicting trends in expression profiles, patient prognosis, and tumor immunology in different types of cancer, which greatly complicated our understanding of the oncogenic role of *SLC6A8*. Therefore, more comprehensive and coherent scientific research on this gene should be conducted.

## Conclusion

To summarize, our study uncovered the expression profile and clinical significance of *SLC6A8* in pan-cancer through bioinformatics analysis for the first time. The aberrant expression of *SLC6A8* predicts poor survival and distinguishes cancer patients from those without cancer in numerous cancers. *SLC6A8* gene is often altered, with amplification being the most common type of alteration. Mechanistically, the results of KEGG and GO analyses showed that *SLC6A8* was significantly enriched in metabolism-associated pathways such as “Carbon metabolism” and “Glycolysis/Gluconeogenesis.” Additionally, *SLC6A8* expression is related to immune infiltration levels, expression of immune checkpoint genes, TMB, and MSI, suggesting the potential of *SLC6A8* for prediction of the efficacy of ICIs.

## Data Availatbility Statenent

The datasets presented in this study can be found in online repositories. The names of the repository/repositories and accession number(s) can be found in the article/Supplementary Material.
